# New biological and genetic classification and therapeutically relevant categories in childhood B-cell precursor acute lymphoblastic leukemia

**DOI:** 10.12688/f1000research.16074.1

**Published:** 2018-09-28

**Authors:** Jan Starý, Jan Zuna, Marketa Zaliova

**Affiliations:** 1 Department of Pediatric Hematology and Oncology, Second Faculty of Medicine, Charles University and University Hospital Motol, Prague, Czech Republic; 2 Childhood Leukaemia Investigation Prague (CLIP), Prague, Czech Republic

**Keywords:** acute lymphoblastic leukemia, children, massive parallel sequencing, new BCP-ALL subtypes

## Abstract

Traditionally, genetic abnormalities detected by conventional karyotyping, fluorescence *in situ* hybridization, and polymerase chain reaction divided childhood B-cell precursor acute lymphoblastic leukemia (BCP-ALL) into well-established genetic subtypes. This genetic classification has been prognostically relevant and thus used for the risk stratification of therapy. Recently, the introduction of genome-wide approaches, including massive parallel sequencing methods (whole-genome, -exome, and -transcriptome sequencing), enabled extensive genomic studies which, together with gene expression profiling, largely expanded our understanding of leukemia pathogenesis and its heterogeneity. Novel BCP-ALL subtypes have been described. Exact identification of recurrent genetic alterations and their combinations facilitates more precise risk stratification of patients. Discovery of targetable lesions in subsets of patients enables the introduction of new treatment modalities into clinical practice and stimulates the transfer of modern methods from research laboratories to routine practice.

## Introduction

Traditionally, B-cell precursor (BCP) acute lymphoblastic leukemia (ALL) has been classified into several distinct genetic subtypes defined by recurrent structural or numerical chromosomal alterations detected by cytogenetic methods (karyotyping and fluorescence *in situ* hybridization [FISH]), polymerase chain reaction (PCR), and flow cytometry (measurement of DNA content corresponding to ploidy) ^1^. Six major genetic subtypes, altogether accounting for 70% to 75% of BCP-ALL, have been defined by high hyperdiploidy (51 to 67 chromosomes per leukemic cell), hypodiploidy (fewer than 45 chromosomes per leukemic cell), and *ETV6-RUNX1*, *TCF3-PBX1*, *BCR-ABL1*, and *KMT2A* gene-involving fusions. These genetic aberrations have been characterized as first leukemogenic hits which are present in all cells comprising the leukemic clone, defining its key biological features as well as impacting the clinical character of respective BCP-ALL subtypes. Their identification has remained important until today for diagnosis specification and, in some of them, for risk classification and targeted therapy ^2^.

Rapid progress of modern genetic techniques yielded important discoveries in various human diseases, including BCP-ALL. Genome-wide profiling using array-based comparative genomic hybridization, single-nucleotide polymorphism arrays, and massive parallel sequencing (MPS) of whole genomes, whole exomes, and whole transcriptomes (RNA sequencing, or RNA-seq) resulted in the identification of novel recurrent genetic aberrations and patterns. Among them, several prognostically significant and druggable aberrations which have started to be implemented into risk-stratification algorithms and targeted therapy have been described ^3^. Moreover, together with genome-wide gene expression profiling on microarrays and by RNA-seq, modern genomic studies led to the identification of novel biologically and clinically relevant BCP-ALL subtypes ^4^. The so-called B-other ALL, a genetically and clinically heterogeneous subset of leukemias accounting for up to 30% of BCP-ALL (herein defined by negativity for all six above-mentioned classifying aberrations), was further dissected and better characterized. This review focuses on recent findings related to five novel BCP-ALL subtypes and selected clinically relevant genetic aberrations/patterns.

## New BCP-ALL subtypes

Similar to the above-mentioned “classical” BCP-ALL subtypes, the five novel subtypes are distinguishable by their gene expression signatures; however, only three of them are also defined by the presence of a subtype-specific genetic aberration.

The first subtype, *BCR-ABL1*-like/Philadelphia chromosome-like (Ph-like) ALL (hereafter *BCR-ABL1*-like ALL), was already described in the “pre-MPS” era thanks to gene expression profiling on microarrays ^5, 6^. It is defined by a gene expression signature similar to that of *BCR-ABL1*-positive ALL. This gene expression signature likely results from the activation of kinase signaling pathways, which, however, is not triggered by a single specific genetic aberration as in the case of *BCR-ABL1*-positive ALL. On the contrary, a very wide spectrum of different genetic aberrations likely inducing this signature has been described in *BCR-ABL1*-like ALL ^7– 11^. The aberrations affect genes encoding kinases (for example, *JAK1*, *JAK2*, *JAK3*, *ABL1*, *ABL2*, and *TYK2*), cytokine and growth factor receptors (for example, *CRLF2*, *PDGFRB*, *EPOR*, *IL7R*, *CSF1R*, *NTRK3*, and *FLT3*), and signaling mediators and regulators ( *KRAS*, *NRAS*, *BRAF*, *PTPN11*, *NF1*, and *SH2B3*). These aberrations can be of various types, including both small-scale (deletions, insertions, substitutions, and complex mutations) and large-scale (chromosomal translocations and deletions) mutations which may generate fusion genes; of note, the majority of kinase/cytokine-receptor genes ( *JAK2*, *ABL1*, and so on) can be fused to several different fusion partners. Such “kinase-signaling aberrations” can be found in 39% to 91% of *BCR-ABL1*-like ALL ^10, 12, 13^; however, some of them also occur in B-other ALL not classified as *BCR-ABL1*-like ^9, 14, 15^.

This ALL subtype was discovered independently by two studies. Although *BCR-ABL1*-like and Ph-like ALL, described by Dutch and American investigators, respectively, are now considered a single subtype, their definition is not fully consistent ^5, 6^. Some patients can be classified discordantly on the basis of classification approaches used by these two studies ^15^. Several factors may contribute to this discrepancy, including methodological differences in gene expression data analysis, different composition of discovery cohorts (with respect to representation of various BCP-ALL subtypes, risk groups, and ethnicities), and the limited specificity of *BCR-ABL1* gene expression signature.

*BCR-ABL1*-like ALL is the only novel subtype that was already added as a provisional entity into the 2016 update of World Health Organization (WHO) ALL classification ^16^. It occurs in about 10% to 15% of pediatric BCP-ALL and accounts for about 15% to 35% of the B-other ALL group and its frequency increases with age. Both above-mentioned *BCR-ABL1*-like discovery studies reported its inferior outcome ^5, 6^. Although recent studies demonstrate that the overall survival of children with Ph-like ALL treated according to protocols employing minimal residual disease (MRD)-based risk stratification is not significantly inferior to that of non- *BCR-ABL1*-like cases, such patients commonly exhibit inferior early response to therapy requiring treatment intensification ^13^. Of note, it has been shown that outcome depends on the genomic context: the type of “kinase aberration” or presence of the *IKZF1* gene deletion (which has been recognized as an unfavorable prognostic factor in BCP-ALL as detailed below) ^10, 17^.

The *ETV6-RUNX1*-like subtype was described within B-other ALL on the basis of tight co-clustering of some *ETV6-RUNX1*-negative cases with *ETV6-RUNX1*-positive ALL according to gene expression profiling ^18^. All cases belonging to this ALL subtype which have been described so far have a common genetic denominator: aberrations of the *ETV6* gene ^18, 19^; however, these are not specific for the *ETV6-RUNX1*-like ALL and occur in other subtypes as well ^18, 19^. The *ETV6* aberrations are usually deletions, but other gene-disrupting structural aberrations that may result in fusion genes were also described. Various alterations of *IKZF1* are also frequently detected within this subtype ^18, 19^. Biological proximity to *ETV6-RUNX1*-positive ALL is further supported by a similar expression pattern of two cell surface markers—CD27 and CD44—which can be measured by flow cytometry. Unlike the vast majority of other BCP-ALL subtypes, both *ETV6-RUNX1*-like and *ETV6-RUNX1*-positive ALL express CD27 but are negative or only partially or weakly positive for CD44 ^19, 20^. Such genotype–phenotype correlation is rather rare in BCP-ALL. Two studies describing this subtype so far indicate that it represents 5% to 12% of B-others and 1% to 3% of all BCP-ALL cases ^18, 19^. *ETV6-RUNX1*-like ALL does not appear to be associated with inferior prognosis; however, additional studies on larger cohorts are needed to reliably determine whether the prognosis of this novel subtype is as favorable as that of *ETV6-RUNX1*-positive ALL ^18, 19^.

*DUX4*-rearranged ALL was originally described as a B-other ALL subset with specific gene expression profile and frequent deletions of the ETS transcription factor gene *ERG* ^21, 22^. Only recently, these leukemias have been characterized by a unique genetic aberration: rearrangements of the gene encoding transcription factor DUX4 ( *DUX4*r) ^18, 23– 25^. These rearrangements are most frequently insertions of *DUX4* into the *IGH* gene, resulting in the *IGH–DUX4* fusion. The expression of *DUX4*, which is physiologically silenced in somatic tissues, is activated in *DUX4r*-ALL by its juxtaposition under the control of an ectopic regulatory element. The deletions of *ERG* can be detected in about 50% to 63% of cases ^18, 25^. Although the rearrangement of *DUX4* is an early, leukemia-initiating event ^24, 25^, the *ERG* deletion is frequently a subclonal, thus secondary, aberration ^26, 27^. It has been demonstrated that DUX4 binds to and deregulates the transcription of *ERG* in *DUX4*r-ALL; it induces the expression of alternative *ERG* variant and perhaps also renders the *ERG* gene prone to deletions ^25^. Owing to the small size of the inserted chromosomal fragment ^18, 24^, the *IGH–DUX4* fusion (and other *DUX4*r) cannot be easily screened by FISH. Similarly, high variability of genomic breakpoints makes a potential fusion screening by PCR challenging. Thus, so far, this ALL subtype has been determined on the basis of its unique expression signature or presence of *DUX4* fusion transcripts detected by RNA-seq ^18, 23– 25^. *DUX4*r-ALL represents 4% to 8% of BCP-ALL and 15% to 30% of B-others. Before the *DUX4*r-ALL discovery, it had been shown that, despite the association with slow early treatment response and with prognostically unfavorable *IKZF1* deletions, *ERG* deletions were associated with favorable outcome ^26, 27^. Two recent studies reported favorable outcome also for *DUX4*r-ALL ^18, 25^. However, although the favorable outcome of BCP-ALL with *ERG* deletions seems to derive from that of the *DUX4*r-ALL subtype, the outcome of *DUX4*r-ALL and potential prognostic impact of *ERG* deletions within this subgroup should be further studied in larger and uniformly treated cohorts of patients. Interestingly, *ERG* deletions have been associated with an aberrant expression of CD2 and the tendency of leukemic blasts to switch immunophenotype from BCP to monocytoid at the beginning of treatment ^27, 28^. During this lineage switch, B lineage or progenitor markers such as CD19 and CD34 (or both) are lost, possibly hampering B-cell-oriented flow cytometric detection of minimal residual disease. Similar to the outcome, the likely association of lineage switch with the *DUX4*r-ALL subtype and impact of the *ERG* deletions on this association remain to be elucidated by future studies.

ALL with the *ZNF384* gene-involving fusions ( *ZNF384*r) represents another novel subtype of BCP-ALL ^23, 24, 29, 30^. The *ZNF384* gene, encoding transcription factor zinc-finger protein 384, can be fused to at least nine different partners (most frequently *TCF3*, *EP300*, or *TAF15*) in BCP-ALL. *ZNF384*r-ALL represents 1% to 5% of BCP-ALL and 5% to 10% of B-others. The unique gene expression signature of this ALL subtype is enriched for hematopoietic stem cell and immature myeloid lineage features and reflects upregulation of the JAK-STAT signaling pathway ^30, 31^. *ZNF384*r-ALL also frequently displays a distinct immunophenotype compared with the majority of other BCP-ALL subtypes; the immunophenotypically distinct *ZNF384*r-ALL cases do not express (or only weakly express) CD10 marker but express myeloid markers CD13 or CD33 (or both) and may be even classified as mixed-phenotype acute leukemias on the basis of EGIL (European Group for the Immunological Characterization of Leukaemias) or WHO criteria ^30, 32– 34^. The clinical features of *ZNF384*r-ALL may vary depending on *ZNF384* fusion partner ^30^. However, based on published studies reporting relatively small numbers of patients so far, *ZNF384*r-ALL does not appear to be a high-risk subtype in children ^30, 34^.

The last of the five novel ALL subtypes is defined by rearrangements of the *MEF2D* gene ( *MEF2D*r) ^23, 24, 29^. So far, at least six distinct *MEF2D*-involving fusion genes (with *BCL9* gene being the most frequent fusion partner) have been described in this ALL subtype ^18, 23, 29^. *MEF2D* encodes a transcription factor that plays a role in muscle and neuronal cell differentiation but is also expressed throughout B-cell differentiation ^35^. The specific gene expression signature suggests a later differentiation stage of *MEF2D*r-ALL compared with other BCP-ALL subtypes ^29^. Immunophenotyping by flow cytometry may also reveal specific features pointing toward this ALL subtype; *MEF2D*r-ALL cells typically have relatively weak expression of CD10 and high expression of CD38 ^29^. *MEF2D*r-ALL represents 1% to 3% of childhood BCP-ALL and 2% to 5% of B-others and is diagnosed more often in adolescents than in children. The outcome of *MEF2Dr-*ALL seems to be inferior to that of other ALL subtypes ^29, 36^. Alternative therapy employing histone deacetylase (HDAC) inhibitors which target *HDAC9* (one of the *MEF2D*r-ALL signature genes) and which were already successfully tested *in vitro* could potentially improve its outcome in the future ^29^.

About 40% of B-other ALL cannot be classified into any of the five novel BCP-ALL subtypes. Various recurrent genomic aberrations can be found in patients belonging to this subset; some of them were already identified decades ago and others more recently. These include, for example, intrachromosomal amplification of chromosome 21 (iAMP21) ^37^, dic(9;20) ^38^, *IGH–MYC* ^39^ and *TCF3–HLF* ^40^ fusions, *CRLF2* ^41, 42^, *NUTM1* ^29^, or *PAX5* ^18, 23, 29^ gene-involving fusions, and intragenic amplification of *PAX5* ( *PAX5*amp) ^43^. Although some of them likely represent primary lesions and could be considered subtype-defining aberrations, others frequently represent secondary aberrations or co-occur with established primary lesions or across established BCP-ALL subtypes. Consequently, certain heterogeneity exists in the classification of BCP-ALL into genetic/biological subtypes throughout the literature as well as in the definition of B-other ALL, which thus should always be explicitly described when used. One example of a potentially confusing situation is the classification of ALL with iAMP21. This ALL subset was added provisionally into the WHO 2016 ALL classification together with *BCR-ABL1*-like ALL ^16^, although it has been shown that these two ALL subsets partly overlap ^15^. Moreover, though it has been suggested to represent a primary lesion ^44^, iAMP21, in a minor proportion of patients, co-occurs with other primary aberrations such as *BCR–ABL1* or *ETV6–RUNX1* fusions. Similarly, *CRLF2* rearrangement ( *CRLF2*r) that is sometimes used to define the subset of patients within B-other ALL ^45^ is frequently a secondary aberration ^46^ and also occurs across several BCP-ALL subtypes ( *BCR-ABL1*-positive ALL, *BCR-ABL1*-like ALL, and hyperdiploid and hypodiploid ALL) ^11, 41, 47, 48^. Nevertheless, identification of these aberrations is clinically relevant, as some of them were proven to have unfavorable prognostic impact and thus influence risk stratification and therapy (for example, iAMP21 ^49, 50^ and *TCF3–HLF* ^51^) whereas in others the suggested prognostic impact needs further validation (for example, *PAX5*amp ^52^). Importantly, various aberrations may qualify patients for a targeted therapy (for example, *CRLF2*r) as further discussed below.

## New therapeutically relevant aberrations/categories

A variety of novel druggable lesions, especially of kinase-activating aberrations, have been described thanks to the introduction of MPS in recent years. As already mentioned above, the kinase-activating lesions are highly enriched in the *BCR-ABL1*-like ALL subtype ^9, 10, 15^. A large proportion of these aberrations can be assigned into one of two functional classes according to the affected signaling pathway: JAK/STAT class and ABL class ^10, 17, 53^. The more frequent JAK/STAT class aberrations comprise aberrations affecting *CRLF2*, *JAK1*, *JAK2*, *JAK3*, *EPOR*, *IL7R*, and *SH2B3* genes and can be targeted by the JAK inhibitor ruxolitinib. The most frequently affected *CRLF2* gene encodes one of two subunits of heterodimeric receptor for thymic stromal lymphopoietin. In addition to constitutively activating F232C mutation ^54, 55^, two types of *CRLF2*-involving fusion have been described: *IGH–CRLF2*, which results from interchromosomal translocation, and *P2RY8–CRLF2*, which is caused by deletion in the PAR1 region on gonosomes ^41, 42^. Both fusions lead to an overexpression of intact CRLF2 protein on the cell surface, which can be reliably detected by flow cytometry ^42, 56^. Thus, with the use of anti-CRLF2 antibody, *CRLF2*r-positive patients (who represent up to 17% of B-other ALL ^18^ and who might be considered for targeted therapy by ruxolitinib) can be easily identified during diagnostic immunophenotyping. The second class of kinase-activating aberrations, the ABL class, comprise *ABL1*, *ABL2*, *PDGFRB*, *PDGFRA*, and *CSF1R* gene-involving fusions. These aberrations can be inhibited by tyrosine-kinase inhibitors (TKIs) such as imatinib or dasatinib ^10^, both of which have already become an inherent component of the treatment of pediatric *BCR-ABL1*-positive ALL ^57^. Aberration of additional receptor and non-receptor kinase genes (for example, *NTRK3*, *TYK2*, *DGKH*, and *PTK2B*) that do not belong to these two classes but that at least in some cases can be targeted by known inhibitors can be also detected, though rarely, in *BCR-ABL1*-like ALL ^10^. In addition to these lesions associated with the *BCR-ABL1*-like phenotype, aberrations resulting in the activation of the Ras/Raf/MAPK pathway (hereafter, Ras pathway) occur frequently across various BCP-ALL subtypes ^58– 62^. Activating point mutations of the *KRAS* and *NRAS* genes are the most abundant, whereas mutations of the *FLT3*, *PTPN11*, *NF1*, and *BRAF* genes occur less frequently. It has been shown that leukemic cells with Ras pathway mutations are sensitive to MEK inhibitors *in vitro* ^63, 64^.

Sensitivity tests performed *in vitro* and in patient-derived xenografts provided a solid rationale for prospective clinical testing of TKIs in BCP-ALL with novel kinase-activating aberrations ^17, 53^. However, a relatively limited number of such children already treated by TKIs have been reported in the literature so far ^10, 14, 65– 73^. Although several case reports described good response to ABL class inhibitors in patients with *ABL1*/ *ABL2* or *PDGFRB* gene-involving fusions ^10, 66, 67, 70– 72, 74^ and overall results are generally encouraging, in some patients the TKI-involving treatment failed to induce long-term remission. This can be at least partially due to the fact that TKIs were added to treatment only upon diagnosis of disease resistance/relapse. Additionally, cases of secondary TKI resistance caused by mutations in targeted kinase (that is, by the most common mechanism of resistance known in *BCR-ABL1*-positive leukemias) have already been described ^75, 76^. The first clinical trials have been initiated recently which use frontline TKIs added to chemotherapy backbone for selected groups of patients with kinase-activating lesions (ClinicalTrials.gov Identifier: NCT03117751 sponsored by St. Jude Children’s Research Hospital, Memphis, TN, USA; ClinicalTrials.gov Identifier: NCT02420717 sponsored by MD Anderson Cancer Center, Houston, TX, USA; ClinicalTrials.gov Identifier: NCT02883049 sponsored by the National Cancer Institute, Rockville, MD, USA; and ClinicalTrials.gov Identifier: NCT03020030 sponsored by the Dana-Farber Cancer Institute, Boston, MA, USA). Importantly, several clinical and biological aspects should be considered in the strategies for prospective TKI testing in newly diagnosed BCP-ALL. It should be discussed carefully whether it is justified to use TKIs in all children with any targetable kinase-activating aberration, whether they should be used only in children harboring a lesion unambiguously associated with unfavorable outcome (similar to *BCR–ABL1* fusion), or whether the TKIs should be reserved for patients with worse early response to treatment or with resistant disease, where the potential benefits most likely outweigh an increase of treatment toxicity ^77^. Moreover, it is important to consider that while some of the kinase and cytokine-receptor gene alterations are supposed to represent founding lesions present in all leukemic cells and essential for their survival ^10, 78– 80^, some aberrations may be either founding or secondary lesions (for example, *CRLF2*r) and others are typically secondary subclonal lesions (for example, *JAK*/ *RAS* gene mutations), possibly without a resistance- or relapse-driving role, thus probably representing less-suitable therapeutic targets ^46, 81^.

Deletions of the *IKZF1* gene ( *IKZF1*del) encoding lymphoid transcription factor Ikaros occur in 9% to 15% of BCP-ALL and more frequently in B-other, *BCR-ABL1*-positive, and hypodiploid ALL compared with remaining subtypes ^17, 43, 59, 82, 83^. In 2009, the *IKZF1*del was associated with poor outcome ^6^; subsequently, multiple studies confirmed its negative prognostic impact in BCP-ALL ^82– 92^. These findings stimulated the incorporation of *IKZF1*del into risk-stratification algorithms as a factor qualifying for treatment intensification in some ALL trials (ClinicalTrials.gov Identifier: NCT03020030 sponsored by the Dana-Farber Cancer Institute and ClinicalTrials.gov Identifier: NCT02716233 sponsored by Assistance Publique - Hôpitaux de Paris, France). However, partially discrepant findings of individual studies showed that the prognostic impact of *IKZF1*del (its strength and independence on other known risk factors) depends on, for example, risk-stratification algorithms, applied therapy, and early treatment response. Moreover, it was revealed that it is modified by the presence of other genetic lesions. First, it was shown that deletions in the *ERG* gene ( *ERG*del) attenuate the negative prognostic impact of *IKZF1*del ^26, 27^; a subsequent study demonstrated that other recurrent deletions may have the opposite effect ^93^. These findings led to the establishment of the *IKZF1*plus category ^93^, defined as *IKZF1*del with concurrent deletion of *PAX5* or *CDKN2A* or *CDKN2B* genes or deletion of PAR1 region (resulting in *P2RY8–CRLF2* fusion gene) or with a combination of these, in the absence of *ERG*del. The *IKZF1*plus genomic pattern occurs in 6% of BCP-ALL, is associated with inferior outcome in patients with detectable minimal residual disease at the end of induction treatment, and will be used for risk stratification in the upcoming AIEOP-BFM (Associazione Italiana di Ematologia ed Oncologia Pediatrica–Berlin-Frankfurt-Münster) ALL trial ^93^. *IKZF1*plus occurs predominantly in B-other ALL; although we can assume its enrichment in subtypes with higher frequency of *IKZF1*del, such as *BCR-ABL1*-like ALL, its distribution and prognostic role across novel BCP-ALL subtypes remain to be elucidated by future studies. These studies could also help to clarify the strong association between *IKZF1*plus prognostic value and the early therapy response.

## Conclusions

Herein, we briefly reviewed some of the most important recent findings in pediatric BCP-ALL, demonstrating that the use of modern high-throughput technologies not only advanced our insight into the genetics and biology of BCP-ALL but also paved the way for novel treatment options. However, with growing knowledge, we have begun to recognize more extensively that the significance (for example, clinical relevance) of individual factors may vary substantially depending on additional genetic/biological contexts. Enormous effort will still be needed to further improve our understanding of the considerably complex relationships between genetic/biological and clinical aspects of BCP-ALL and to better translate this knowledge into further improvements in patient outcome.

## Abbreviations

ALL, acute lymphoblastic leukemia; BCP, B-cell precursor; FISH, fluorescence *in situ* hybridization; MPS, massive parallel sequencing; PCR, polymerase chain reaction; Ph-like, Philadelphia chromosome-like; RNA-seq, RNA sequencing; TKI, tyrosine-kinase inhibitor; WHO, World Health Organization

## References

[1] Pui CH Relling MV Downing JR: Acute lymphoblastic leukemia. *N Engl J Med.* 2004;350(15):1535–48. 10.1056/NEJMra023001 15071128

[2] Harrison CJ Haas O Harbott J: Detection of prognostically relevant genetic abnormalities in childhood B-cell precursor acute lymphoblastic leukaemia: recommendations from the Biology and Diagnosis Committee of the International Berlin-Frankfürt-Münster study group. *Br J Haematol.* 2010;151(2):132–42. 10.1111/j.1365-2141.2010.08314.x 20701601

[3] Iacobucci I Mullighan CG: Genetic Basis of Acute Lymphoblastic Leukemia. *J Clin Oncol.* 2017;35(9):975–83. 10.1200/JCO.2016.70.7836 28297628 PMC5455679

[4] Lilljebjörn H Fioretos T: New oncogenic subtypes in pediatric B-cell precursor acute lymphoblastic leukemia. *Blood.* 2017;130(12):1395–401. 10.1182/blood-2017-05-742643 28778863

[5] Den Boer ML van Slegtenhorst M De Menezes RX: A subtype of childhood acute lymphoblastic leukaemia with poor treatment outcome: a genome-wide classification study. *Lancet Oncol.* 2009;10(2):125–34. 10.1016/S1470-2045(08)70339-5 19138562 PMC2707020

[6] Mullighan CG Su X Zhang J: Deletion of *IKZF1* and prognosis in acute lymphoblastic leukemia. *N Engl J Med.* 2009;360(5):470–80. 10.1056/NEJMoa0808253 19129520 PMC2674612

[7] Boer JM Steeghs EM Marchante JR: Tyrosine kinase fusion genes in pediatric *BCR-ABL1*-like acute lymphoblastic leukemia. *Oncotarget.* 2017;8(3):4618–28. 10.18632/oncotarget.13492 27894077 PMC5354859

[8] Imamura T Kiyokawa N Kato M: Characterization of pediatric Philadelphia-negative B-cell precursor acute lymphoblastic leukemia with kinase fusions in Japan. *Blood Cancer J.* 2016;6:e419. 10.1038/bcj.2016.28 27176795 PMC4916297

[9] Reshmi SC Harvey RC Roberts KG: Targetable kinase gene fusions in high-risk B-ALL: a study from the Children's Oncology Group. *Blood.* 2017;129(25):3352–61. 10.1182/blood-2016-12-758979 28408464 PMC5482101

[10] Roberts KG Li Y Payne-Turner D: Targetable kinase-activating lesions in Ph-like acute lymphoblastic leukemia. *N Engl J Med.* 2014;371(11):1005–15. 10.1056/NEJMoa1403088 25207766 PMC4191900

[11] Roberts KG Morin RD Zhang J: Genetic alterations activating kinase and cytokine receptor signaling in high-risk acute lymphoblastic leukemia. *Cancer Cell.* 2012;22(2):153–66. 10.1016/j.ccr.2012.06.005 22897847 PMC3422513

[12] Roberts KG Reshmi SC Harvey RC: Genomic and outcome analyses of Ph-like ALL in NCI standard-risk patients: a report from the Children's Oncology Group. *Blood.* 2018;132(8):815–24. 10.1182/blood-2018-04-841676 29997224 PMC6107876

[13] Roberts KG Pei D Campana D: Outcomes of children with *BCR-ABL1*–like acute lymphoblastic leukemia treated with risk-directed therapy based on the levels of minimal residual disease. *J Clin Oncol.* 2014;32(27):3012–20. 10.1200/JCO.2014.55.4105 25049327 PMC4162497

[14] Zaliova M Moorman AV Cazzaniga G: Characterization of leukemias with *ETV6-ABL1* fusion. *Haematologica.* 2016;101(9):1082–93. 10.3324/haematol.2016.144345 27229714 PMC5060025

[15] Boer JM Marchante JR Evans WE: *BCR-ABL1*-like cases in pediatric acute lymphoblastic leukemia: a comparison between DCOG/Erasmus MC and COG/St. Jude signatures. *Haematologica.* 2015;100(9):e354–7. 10.3324/haematol.2015.124941 26045294 PMC4800707

[16] Arber DA Orazi A Hasserjian R: The 2016 revision to the World Health Organization classification of myeloid neoplasms and acute leukemia. *Blood.* 2016;127(20):2391–405. 10.1182/blood-2016-03-643544 27069254

[17] Roberts KG Yang YL Payne-Turner D: Oncogenic role and therapeutic targeting of ABL-class and JAK-STAT activating kinase alterations in Ph-like ALL. *Blood Adv.* 2017;1(20):1657–71. 29296813 10.1182/bloodadvances.2017011296 PMC5728345

[18] Lilljebjörn H Henningsson R Hyrenius-Wittsten A: Identification of *ETV6-RUNX1*-like and *DUX4*-rearranged subtypes in paediatric B-cell precursor acute lymphoblastic leukaemia. *Nat Commun.* 2016;7:11790. 10.1038/ncomms11790 27265895 PMC4897744

[19] Zaliova M Kotrova M Bresolin S: *ETV6/RUNX1*-like acute lymphoblastic leukemia: A novel B-cell precursor leukemia subtype associated with the CD27/CD44 immunophenotype. *Genes Chromosomes Cancer.* 2017;56(8):608–16. 10.1002/gcc.22464 28395118

[20] Vaskova M Mejstrikova E Kalina T: Transfer of genomics information to flow cytometry: expression of CD27 and CD44 discriminates subtypes of acute lymphoblastic leukemia. *Leukemia.* 2005;19(5):876–8. 10.1038/sj.leu.2403706 15759032

[21] Mullighan CG Miller CBS Su X: *ERG* Deletions Define a Novel Subtype of B-Progenitor Acute Lymphoblastic Leukemia. *Blood.*(ASH Annual Meeting Abstracts).2007;110(11):691 Reference Source

[22] Yeoh EJ Ross ME Shurtleff SA: Classification, subtype discovery, and prediction of outcome in pediatric acute lymphoblastic leukemia by gene expression profiling. *Cancer Cell.* 2002;1(2):133–43. 10.1016/S1535-6108(02)00032-6 12086872

[23] Liu YF Wang BY Zhang WN: Genomic Profiling of Adult and Pediatric B-cell Acute Lymphoblastic Leukemia. *EBioMedicine.* 2016;8:173–83. 10.1016/j.ebiom.2016.04.038 27428428 PMC4919728

[24] Yasuda T Tsuzuki S Kawazu M: Recurrent *DUX4* fusions in B cell acute lymphoblastic leukemia of adolescents and young adults. *Nat Genet.* 2016;48(5):569–74. 10.1038/ng.3535 27019113

[25] Zhang J McCastlain K Yoshihara H: Deregulation of *DUX4* and ERG in acute lymphoblastic leukemia. *Nat Genet.* 2016;48(12):1481–9. 10.1038/ng.3691 27776115 PMC5144107

[26] Clappier E Auclerc MF Rapion J: An intragenic *ERG* deletion is a marker of an oncogenic subtype of B-cell precursor acute lymphoblastic leukemia with a favorable outcome despite frequent *IKZF1* deletions. *Leukemia.* 2014;28(1):70–7. 10.1038/leu.2013.277 24064621

[27] Zaliova M Zimmermannova O Dörge P: *ERG* deletion is associated with CD2 and attenuates the negative impact of *IKZF1* deletion in childhood acute lymphoblastic leukemia. *Leukemia.* 2014;28(1):182–5. 10.1038/leu.2013.282 24072102

[28] Slamova L Starkova J Fronkova E: CD2-positive B-cell precursor acute lymphoblastic leukemia with an early switch to the monocytic lineage. *Leukemia.* 2014;28(3):609–20. 10.1038/leu.2013.354 24270736

[29] Gu Z Churchman M Roberts K: Genomic analyses identify recurrent *MEF2D* fusions in acute lymphoblastic leukaemia. *Nat Commun.* 2016;7:13331. 10.1038/ncomms13331 27824051 PMC5105166

[30] Hirabayashi S Ohki K Nakabayashi K: *ZNF384*-related fusion genes define a subgroup of childhood B-cell precursor acute lymphoblastic leukemia with a characteristic immunotype. *Haematologica.* 2017;102(1):118–29. 10.3324/haematol.2016.151035 27634205 PMC5210242

[31] McClure BJ Heatley SL Kok CH: Pre-B acute lymphoblastic leukaemia recurrent fusion, *EP300-ZNF384*, is associated with a distinct gene expression. *Br J Cancer.* 2018;118(7):1000–4. 10.1038/s41416-018-0022-0 29531323 PMC5931087

[32] Gocho Y Kiyokawa N Ichikawa H: A novel recurrent *EP300-ZNF384* gene fusion in B-cell precursor acute lymphoblastic leukemia. *Leukemia.* 2015;29(12):2445–8. 10.1038/leu.2015.111 25943178

[33] Hrusak O de Haas V Stancikova J: International cooperative study identifies treatment strategy in childhood ambiguous lineage leukemia. *Blood.* 2018;132(3):264–76. 10.1182/blood-2017-12-821363 29720486

[34] Shago M Abla O Hitzler J: Frequency and outcome of pediatric acute lymphoblastic leukemia with *ZNF384* gene rearrangements including a novel translocation resulting in an *ARID1B/ZNF384* gene fusion. *Pediatr Blood Cancer.* 2016;63(11):1915–21. 10.1002/pbc.26116 27392123

[35] Herglotz J Unrau L Hauschildt F: Essential control of early B-cell development by Mef2 transcription factors. *Blood.* 2016;127(5):572–81. 10.1182/blood-2015-04-643270 26660426

[36] Suzuki K Okuno Y Kawashima N: *MEF2D-BCL9* Fusion Gene Is Associated With High-Risk Acute B-Cell Precursor Lymphoblastic Leukemia in Adolescents. *J Clin Oncol.* 2016;34(28):3451–9. 10.1200/JCO.2016.66.5547 27507882

[37] Harewood L Robinson H Harris R: Amplification of *AML1* on a duplicated chromosome 21 in acute lymphoblastic leukemia: a study of 20 cases. *Leukemia.* 2003;17(3):547–53. 10.1038/sj.leu.2402849 12646943

[38] Slater R Smit E Kroes W: A non-random chromosome abnormality found in precursor-B lineage acute lymphoblastic leukaemia: dic(9;20)(p1?3;q11). *Leukemia.* 1995;9(10):1613–9. 7564498

[39] Russell LJ Enshaei A Jones L: *IGH*@ translocations are prevalent in teenagers and young adults with acute lymphoblastic leukemia and are associated with a poor outcome. *J Clin Oncol.* 2014;32(14):1453–62. 10.1200/JCO.2013.51.3242 24711557

[40] Hunger SP Ohyashiki K Toyama K: Hlf, a novel hepatic bZIP protein, shows altered DNA-binding properties following fusion to E2A in t(17;19) acute lymphoblastic leukemia. *Genes Dev.* 1992;6(9):1608–20. 10.1101/gad.6.9.1608 1516826

[41] Mullighan CG Collins-Underwood JR Phillips LA: Rearrangement of *CRLF2* in B-progenitor- and Down syndrome-associated acute lymphoblastic leukemia. *Nat Genet.* 2009;41(11):1243–6. 10.1038/ng.469 19838194 PMC2783810

[42] Russell LJ Capasso M Vater I: Deregulated expression of cytokine receptor gene, *CRLF2*, is involved in lymphoid transformation in B-cell precursor acute lymphoblastic leukemia. *Blood.* 2009;114(13):2688–98. 10.1182/blood-2009-03-208397 19641190

[43] Mullighan CG Goorha S Radtke I: Genome-wide analysis of genetic alterations in acute lymphoblastic leukaemia. *Nature.* 2007;446(7137):758–64. 10.1038/nature05690 17344859

[44] Rand V Parker H Russell LJ: Genomic characterization implicates iAMP21 as a likely primary genetic event in childhood B-cell precursor acute lymphoblastic leukemia. *Blood.* 2011;117(25):6848–55. 10.1182/blood-2011-01-329961 21527530

[45] Hunger SP Mullighan CG: Redefining ALL classification: toward detecting high-risk ALL and implementing precision medicine. *Blood.* 2015;125(26):3977–87. 10.1182/blood-2015-02-580043 25999453 PMC4481590

[46] Morak M Attarbaschi A Fischer S: Small sizes and indolent evolutionary dynamics challenge the potential role of *P2RY8-CRLF2*-harboring clones as main relapse-driving force in childhood ALL. *Blood.* 2012;120(26):5134–42. 10.1182/blood-2012-07-443218 23091296 PMC4194314

[47] Ensor HM Schwab C Russell LJ: Demographic, clinical, and outcome features of children with acute lymphoblastic leukemia and *CRLF2* deregulation: results from the MRC ALL97 clinical trial. *Blood.* 2011;117(7):2129–36. 10.1182/blood-2010-07-297135 21106984

[48] Jain N Lu X Daver N: Co-occurrence of CRLF2-rearranged and Ph+ acute lymphoblastic leukemia: a report of four patients. *Haematologica.* 2017;102(12):e514–e517. 10.3324/haematol.2016.161000 28860345 PMC5709127

[49] Heerema NA Carroll AJ Devidas M: Intrachromosomal amplification of chromosome 21 is associated with inferior outcomes in children with acute lymphoblastic leukemia treated in contemporary standard-risk children's oncology group studies: a report from the children's oncology group. *J Clin Oncol.* 2013;31(27):3397–402. 10.1200/JCO.2013.49.1308 23940221 PMC3770866

[50] Moorman AV Robinson H Schwab C: Risk-directed treatment intensification significantly reduces the risk of relapse among children and adolescents with acute lymphoblastic leukemia and intrachromosomal amplification of chromosome 21: a comparison of the MRC ALL97/99 and UKALL2003 trials. *J Clin Oncol.* 2013;31(27):3389–96. 10.1200/JCO.2013.48.9377 23940220

[51] Fischer U Forster M Rinaldi A: Genomics and drug profiling of fatal *TCF3-HLF*-positive acute lymphoblastic leukemia identifies recurrent mutation patterns and therapeutic options. *Nat Genet.* 2015;47(9):1020–9. 10.1038/ng.3362 26214592 PMC4603357

[52] Schwab C Nebral K Chilton L: Intragenic amplification of *PAX5*: a novel subgroup in B-cell precursor acute lymphoblastic leukemia? *Blood Adv.* 2017;1(19):1473–7. 10.1182/bloodadvances.2017006734 29296789 PMC5728462

[53] Boer JM den Boer ML: *BCR-ABL1*-like acute lymphoblastic leukaemia: From bench to bedside. *Eur J Cancer.* 2017;82:203–18. 10.1016/j.ejca.2017.06.012 28709134

[54] Yoda A Yoda Y Chiaretti S: Functional screening identifies CRLF2 in precursor B-cell acute lymphoblastic leukemia. *Proc Natl Acad Sci U S A.* 2010;107(1):252–7. 10.1073/pnas.0911726107 20018760 PMC2806782

[55] Hertzberg L Vendramini E Ganmore I: Down syndrome acute lymphoblastic leukemia, a highly heterogeneous disease in which aberrant expression of *CRLF2* is associated with mutated *JAK2*: a report from the International BFM Study Group. *Blood.* 2010;115(5):1006–17. 10.1182/blood-2009-08-235408 19965641

[56] Bugarin C Sarno J Palmi C: Fine tuning of surface CRLF2 expression and its associated signaling profile in childhood B-cell precursor acute lymphoblastic leukemia. *Haematologica.* 2015;100(6):e229–32. 10.3324/haematol.2014.114447 25862705 PMC4450636

[57] Biondi A Schrappe M De Lorenzo P: Imatinib after induction for treatment of children and adolescents with Philadelphia-chromosome-positive acute lymphoblastic leukaemia (EsPhALL): a randomised, open-label, intergroup study. *Lancet Oncol.* 2012;13(9):936–45. 10.1016/S1470-2045(12)70377-7 22898679 PMC3431502

[58] Andersson AK Ma J Wang J: The landscape of somatic mutations in infant *MLL*-rearranged acute lymphoblastic leukemias. *Nat Genet.* 2015;47(4):330–7. 10.1038/ng.3230 25730765 PMC4553269

[59] Holmfeldt L Wei L Diaz-Flores E: The genomic landscape of hypodiploid acute lymphoblastic leukemia. *Nat Genet.* 2013;45(3):242–52. 10.1038/ng.2532 23334668 PMC3919793

[60] Paulsson K Lilljebjörn H Biloglav A: The genomic landscape of high hyperdiploid childhood acute lymphoblastic leukemia. *Nat Genet.* 2015;47(6):672–6. 10.1038/ng.3301 25961940

[61] Zhang J Mullighan CG Harvey RC: Key pathways are frequently mutated in high-risk childhood acute lymphoblastic leukemia: a report from the Children's Oncology Group. *Blood.* 2011;118(11):3080–7. 10.1182/blood-2011-03-341412 21680795 PMC3175785

[62] Zaliova M Hovorkova L Vaskova M: Slower early response to treatment and distinct expression profile of childhood high hyperdiploid acute lymphoblastic leukaemia with DNA index < 1.16. *Genes Chromosomes Cancer.* 2016;55(9):727–37. 10.1002/gcc.22374 27163296

[63] Irving J Matheson E Minto L: Ras pathway mutations are prevalent in relapsed childhood acute lymphoblastic leukemia and confer sensitivity to MEK inhibition. *Blood.* 2014;124(23):3420–30. 10.1182/blood-2014-04-531871 25253770 PMC4246039

[64] Jerchel IS Hoogkamer AQ Ariës IM: RAS pathway mutations as a predictive biomarker for treatment adaptation in pediatric B-cell precursor acute lymphoblastic leukemia. *Leukemia.* 2018;32(4):931–40. 10.1038/leu.2017.303 28972594 PMC5886052

[65] Duployez N Grzych G Ducourneau B: *NUP214-ABL1* fusion defines a rare subtype of B-cell precursor acute lymphoblastic leukemia that could benefit from tyrosine kinase inhibitors. *Haematologica.* 2016;101(4):e133–4. 10.3324/haematol.2015.136499 26681761 PMC5004396

[66] Kobayashi K Miyagawa N Mitsui K: TKI dasatinib monotherapy for a patient with Ph-like ALL bearing *ATF7IP/PDGFRB* translocation. *Pediatr Blood Cancer.* 2015;62(6):1058–60. 10.1002/pbc.25327 25400122

[67] Lengline E Beldjord K Dombret H: Successful tyrosine kinase inhibitor therapy in a refractory B-cell precursor acute lymphoblastic leukemia with *EBF1-PDGFRB* fusion. *Haematologica.* 2013;98(11):e146–8. 10.3324/haematol.2013.095372 24186319 PMC3815191

[68] Masuzawa A Kiyotani C Osumi T: Poor responses to tyrosine kinase inhibitors in a child with precursor B-cell acute lymphoblastic leukemia with *SNX2-ABL1* chimeric transcript. *Eur J Haematol.* 2014;92(3):263–7. 10.1111/ejh.12234 24215620

[69] Mayfield JR Czuchlewski DR Gale JM: Integration of ruxolitinib into dose-intensified therapy targeted against a novel *JAK2* F694L mutation in B-precursor acute lymphoblastic leukemia. *Pediatr Blood Cancer.* 2017;64(5):e26328. 10.1002/pbc.26328 27860260 PMC5366086

[70] Perwein T Strehl S König M: Imatinib-induced long-term remission in a relapsed *RCSD1-ABL1*-positive acute lymphoblastic leukemia. *Haematologica.* 2016;101(8):e332–5. 10.3324/haematol.2015.139568 27125982 PMC4967583

[71] Schwab C Ryan SL Chilton L: *EBF1-PDGFRB* fusion in pediatric B-cell precursor acute lymphoblastic leukemia (BCP-ALL): genetic profile and clinical implications. *Blood.* 2016;127(18):2214–8. 10.1182/blood-2015-09-670166 26872634

[72] Weston BW Hayden MA Roberts KG: Tyrosine kinase inhibitor therapy induces remission in a patient with refractory *EBF1-PDGFRB*-positive acute lymphoblastic leukemia. *J Clin Oncol.* 2013;31(25):e413–6. 10.1200/JCO.2012.47.6770 23835704

[73] Ding YY Stern JW Jubelirer TF: Clinical efficacy of ruxolitinib and chemotherapy in a child with Philadelphia chromosome-like acute lymphoblastic leukemia with *GOLGA5-JAK2* fusion and induction failure. *Haematologica.* 2018;103(9):e427–e431. 10.3324/haematol.2018.192088 29773603 PMC6119161

[74] Malone A Langabeer S O'Marcaigh A: A doctor(s) dilemma: ETV6-ABL1 positive acute lymphoblastic leukaemia. *Br J Haematol.* 2010;151(1):101–2. 10.1111/j.1365-2141.2010.08323.x 20618334

[75] Yeung DT Moulton DJ Heatley SL: Relapse of *BCR-ABL1*-like ALL mediated by the *ABL1* kinase domain mutation T315I following initial response to dasatinib treatment. *Leukemia.* 2015;29(1):230–2. 10.1038/leu.2014.256 25179732

[76] Zhang Y Gao Y Zhang H: *PDGFRB* mutation and tyrosine kinase inhibitor resistance in Ph-like acute lymphoblastic leukemia. *Blood.* 2018;131(20):2256–61. 10.1182/blood-2017-11-817510 29434033 PMC5958655

[77] Slayton WB Schultz KR Kairalla JA: Dasatinib Plus Intensive Chemotherapy in Children, Adolescents, and Young Adults With Philadelphia Chromosome-Positive Acute Lymphoblastic Leukemia: Results of Children's Oncology Group Trial AALL0622. *J Clin Oncol.* 2018;36(22):2306–14. 10.1200/JCO.2017.76.7228 29812996 PMC6067800

[78] Lukes J Jr Potuckova E Sramkova L: Two novel fusion genes, *AIF1L-ETV6* and *ABL1-AIF1L*, result together with *ETV6-ABL1* from a single chromosomal rearrangement in acute lymphoblastic leukemia with prenatal origin. *Genes Chromosomes Cancer.* 2018;57(9):471–7. 10.1002/gcc.6 29726059

[79] Zimmermannova O Doktorova E Stuchly J: An activating mutation of *GNB1* is associated with resistance to tyrosine kinase inhibitors in *ETV6-ABL1*-positive leukemia. *Oncogene.* 2017;36(43):5985–94. 10.1038/onc.2017.210 28650474 PMC5666322

[80] Zuna J Zaliova M Muzikova K: Acute leukemias with *ETV6/ABL1* ( *TEL/ABL*) fusion: poor prognosis and prenatal origin. *Genes Chromosomes Cancer.* 2010;49(10):873–84. 10.1002/gcc.20796 20589932

[81] Schwartzman O Savino AM Gombert M: Suppressors and activators of JAK-STAT signaling at diagnosis and relapse of acute lymphoblastic leukemia in Down syndrome. *Proc Natl Acad Sci U S A.* 2017;114(20):E4030–E4039. 10.1073/pnas.1702489114 28461505 PMC5441776

[82] Clappier E Grardel N Bakkus M: *IKZF1* deletion is an independent prognostic marker in childhood B-cell precursor acute lymphoblastic leukemia, and distinguishes patients benefiting from pulses during maintenance therapy: results of the EORTC Children's Leukemia Group study 58951. *Leukemia.* 2015;29(11):2154–61. 10.1038/leu.2015.134 26050650

[83] van der Veer A Zaliova M Mottadelli F: *IKZF1* status as a prognostic feature in *BCR-ABL1*-positive childhood ALL. *Blood.* 2014;123(11):1691–8. 10.1182/blood-2013-06-509794 24366361

[84] Asai D Imamura T Suenobu S: *IKZF1* deletion is associated with a poor outcome in pediatric B-cell precursor acute lymphoblastic leukemia in Japan. *Cancer Med.* 2013;2(3):412–9. 10.1002/cam4.87 23930217 PMC3699852

[85] Chen IM Harvey RC Mullighan CG: Outcome modeling with *CRLF2, IKZF1, JAK*, and minimal residual disease in pediatric acute lymphoblastic leukemia: a Children's Oncology Group study. *Blood.* 2012;119(15):3512–22. 10.1182/blood-2011-11-394221 22368272 PMC3325039

[86] Dörge P Meissner B Zimmermann M: *IKZF1* deletion is an independent predictor of outcome in pediatric acute lymphoblastic leukemia treated according to the ALL-BFM 2000 protocol. *Haematologica.* 2013;98(3):428–32. 10.3324/haematol.2011.056135 22875627 PMC3659952

[87] Kuiper RP Waanders E van der Velden VH: *IKZF1* deletions predict relapse in uniformly treated pediatric precursor B-ALL. *Leukemia.* 2010;24(7):1258–64. 10.1038/leu.2010.87 20445578

[88] Olsson L Castor A Behrendtz M: Deletions of *IKZF1* and *SPRED1* are associated with poor prognosis in a population-based series of pediatric B-cell precursor acute lymphoblastic leukemia diagnosed between 1992 and 2011. *Leukemia.* 2014;28(2):302–10. 10.1038/leu.2013.206 23823658

[89] Olsson L Ivanov Öfverholm I Norén-Nyström U: The clinical impact of *IKZF1* deletions in paediatric B-cell precursor acute lymphoblastic leukaemia is independent of minimal residual disease stratification in Nordic Society for Paediatric Haematology and Oncology treatment protocols used between 1992 and 2013. *Br J Haematol.* 2015;170(6):847–58. 10.1111/bjh.13514 26018335

[90] van der Veer A Waanders E Pieters R: Independent prognostic value of *BCR-ABL1-like* signature and *IKZF1* deletion, but not high *CRLF2* expression, in children with B-cell precursor ALL. *Blood.* 2013;122(15):2622–9. 10.1182/blood-2012-10-462358 23974192 PMC3795461

[91] Yamashita Y Shimada A Yamada T: *IKZF1* and *CRLF2* gene alterations correlate with poor prognosis in Japanese *BCR-ABL1*-negative high-risk B-cell precursor acute lymphoblastic leukemia. *Pediatr Blood Cancer.* 2013;60(10):1587–92. 10.1002/pbc.24571 23804397

[92] Yang YL Hung CC Chen JS: *IKZF1* deletions predict a poor prognosis in children with B-cell progenitor acute lymphoblastic leukemia: a multicenter analysis in Taiwan. *Cancer Sci.* 2011;102(10):1874–81. 10.1111/j.1349-7006.2011.02031.x 21740479 PMC11159039

[93] Stanulla M Dagdan E Zaliova M: *IKZF1* ^plus^ Defines a New Minimal Residual Disease-Dependent Very-Poor Prognostic Profile in Pediatric B-Cell Precursor Acute Lymphoblastic Leukemia. *J Clin Oncol.* 2018;36(12):1240–9. 10.1200/JCO.2017.74.3617 29498923

